# M1 Bone Marrow-Derived Macrophage-Derived Extracellular Vesicles Inhibit Angiogenesis and Myocardial Regeneration Following Myocardial Infarction via the MALAT1/MicroRNA-25-3p/CDC42 Axis

**DOI:** 10.1155/2021/9959746

**Published:** 2021-10-29

**Authors:** Bairong Chen, Liyun Luo, Xiaoliang Wei, Dong Gong, Zhihui Li, Songbiao Li, Wenyi Tang, Lizi Jin

**Affiliations:** ^1^Departments of Cardiovascular Disease IV, The Fifth Affiliated Hospital of Sun Yat-sen University, China; ^2^Departments of Cardiovascular Disease I, The Fifth Affiliated Hospital of Sun Yat-sen University, China; ^3^Departments of Cardiovascular Disease III, The Fifth Affiliated Hospital of Sun Yat-sen University, China

## Abstract

Myocardial infarction (MI) is a severe cardiovascular disease. Some M1 macrophage-derived extracellular vesicles (EVs) are involved in the inhibition of angiogenesis and acceleration dysfunction during MI. However, the potential mechanism of M1 phenotype bone marrow-derived macrophages- (BMMs-) EVs (M1-BMMs-EVs) in MI is largely unknown. This study sought to investigate whether M1-BMMs-EVs increased CDC42 expression and activated the MEK/ERK pathway by carrying lncRNA MALAT1 and competitively binding to miR-25-3p, thus inhibiting angiogenesis and myocardial regeneration after MI. After EV treatment, the cardiac function, infarct size, fibrosis, angiogenesis, and myocardial regeneration of MI mice and the viability, proliferation and angiogenesis of oxygen-glucose deprivation- (OGD-) treated myocardial microvascular endothelial cells (MMECs) were assessed. MALAT1 expression in MI mice, cells, and EVs was detected. MALAT1 downstream microRNAs (miRs), genes, and pathways were predicted and verified. MALAT1 and miR-25-3p were intervened to evaluate EV effects on OGD-treated cells. In MI mice, EV treatment aggravated MI and inhibited angiogenesis and myocardial regeneration. In OGD-treated cells, EV treatment suppressed cell viability, proliferation, and angiogenesis. MALAT1 was highly expressed in MI mice, OGD-treated MMECs, M1-BMMs, and EVs. Silencing MALAT1 weakened the inhibition of EV treatment on OGD-treated cells. MALAT1 sponged miR-25-3p to upregulate CDC42. miR-25-3p overexpression promoted OGD-treated cell viability, proliferation, and angiogenesis. The MEK/ERK pathway was activated after EV treatment. Collectively, M1-BMMs-EVs inhibited angiogenesis and myocardial regeneration following MI via the MALAT1/miR-25-3p/CDC42 axis and the MEK/ERK pathway activation.

## 1. Introduction

Myocardial infarction (MI), a chief cause of cardiovascular disease-related death, is characterized by cardiac blood flow interruption with a high incidence rate and mortality [1–3]. MI seriously affects the life span and life quality of the world population and brings enormous pressure and challenges to the global medical system [4–7]. MI is generally a consequence of coronary thrombosis evolved from atherosclerotic plaque rupture and myocardial ischemia-reperfusion injury [8]. In the first 1-2 weeks after MI, myocardial tissues come through rapid changes, including extracellular matrix digestion and fibrosis [9]. Cardiac repair after MI involving myocardial regeneration and angiogenesis is essential for the recovery of impaired cardiac function in MI [10, 11]. Currently, although standard therapies including timely revascularization of ischemic myocardium help to decrease the acute mortality following MI, the long-term prognosis of MI is still poor [9]. Therefore, it is of great significance to explore novel approaches for more effective treatments of MI.

Macrophages have been demonstrated to show a close involvement in the pathogenesis of MI and tissue repair after MI [12, 13]. As the core regulatory cell population of inflammatory responses, macrophages play an important role in the process of ventricular remodeling after MI [14–16]. Bone marrow-derived macrophages (BMMs) are implicated in the early “inflammatory” stage of MI and participate in the subsequent cardiac repair and remodeling following MI [17]. Additionally, the polarization of BMMs (proinflammatory M1-type and anti-inflammatory M2-type) is tightly associated with cardiac repair and regeneration after MI [1, 18]. M1-type BMMs (M1-BMMs) are related to the inhibited angiogenesis, ischemic muscle blood flow reconstruction, and perfusion recovery, contributing to atherosclerotic cardiovascular disease including MI [19, 20].

Recently, extracellular vesicles (EVs), known as cell-derived membrane structures mediating intercellular communication, have been evidenced to play a critical role in the pathology of cardiovascular diseases including MI [21–23]. A previous study has verified that M1 macrophage-derived EVs suppress angiogenesis and exacerbate cardiac dysfunction during MI [24]. In the process of atherosclerosis, M1-BMMs-derived EVs (M1-BMMs-EVs) promote the proliferation and migration of vascular smooth muscle cells, thus aggravating vascular injury [24, 25]. Nevertheless, the underlying mechanism of M1-BMMs-EVs in MI remains largely unknown.

It is well established that EVs can transport their cargoes including long noncoding RNAs (lncRNAs) between cells, to affect the functions and behaviors of the recipient cells [26, 27]. lncRNAs have been validated to show differential expression in MI [28]. In a prior study, lncRNA metastasis-associated lung adenocarcinoma transcript 1 (MALAT1) is proposed as a potential biomarker of MI [29].

Therefore, it is reasonable to speculate that M1-BMMs-EVs play a role in MI with the involvement of MALAT1. Consequently, we performed a series of histological and molecular experiments to identify the role and mechanism of M1-BMMs-EVs in MI, with the purpose to provide some novel therapies against MI.

## 2. Materials and Methods

### 2.1. Ethics Statement

This study was performed with the approval of the Clinical Ethical Committee of The Fifth Affiliated Hospital of Sun Yat-sen University. All procedures were strictly conducted in accordance with the Guide for the Care and Use of Laboratory Animals according to the regulation of People's Republic of China and National Institutes of Health (NIH Publication No. 85-23, revised 1996). The animal experiments were conducted based on the minimized animal number and the least pain.

### 2.2. Induction and Identification of MI-BMMs

Primary BMMs were isolated from bone marrow. In short, C57BL/6J mice (aged 6 weeks, male and female) supplied by Beijing HFK Biotechnology Co., Ltd., Beijing, China (SCXK (Beijing) 2019-0008)), were euthanized quickly after deep anesthesia. The bone marrow extracted from the femur and tibia were placed in the *α*-minimal essential medium (containing 10% inactivated fetal bovine serum (FBS), 100 IU/mL penicillin, and 100 *μ*g/mL streptomycin) (PeproTech, Rocky Hill, NJ, USA) and chopped meat glucose medium (conditioned medium with 100 ng/mL mouse macrophage-colony stimulating factor) (PeproTech) at a ratio of 1 : 10. Following 3-day incubation (5% CO_2_, 37°C), the cells were rinsed for depleting the remaining stromal cells when the medium was changed. Upon 90% confluence, the cells underwent 3 washes with phosphate-buffered saline (PBS) and then 30 min treatment with trypsin. Next, the nonadherent cells were stratified to Ficoll density gradient solution and centrifuged (440 g, 30 min) at room temperature. The upper cells were collected as BMMs. The harvested BMMs were subjected to 36-h polarization stimulation by the addition of an appropriate amount of lipopolysaccharide (LPS; Sigma-Aldrich, Merck KGaA, Darmstadt, Germany) at a final concentration of 100 ng/mL and cytokine interferon-*γ* (IFN-*γ*; PeproTech) at a final concentration of 20 ng/mL into the cell culture medium. Afterward, M1-BMMs were obtained. BMMs and M1-BMMs were observed and photographed under a scanning electron microscope (Hitachi High-Technologies Corp., Tokyo, Japan) and an inverted microscope (Olympus Optical Co., Ltd., Tokyo, Japan). BMMs markers F4/80 (ab105155, Abcam Inc., Cambridge, MA, USA), cluster of differentiation (CD)11b (ab24874, Abcam), and CD11c (ab210308, Abcam) were detected by flow cytometry. BMMs and M1-BMMs were identified using immunofluorescence.

### 2.3. Immunofluorescence

BMMs and M1-BMMs were seeded into 24-well plates, respectively, washed with PBS, and fixed in 4% paraformaldehyde (Beyotime Biotechnology Co., Ltd., Shanghai, China). Next, the cells were permeated with 0.2% Triton X-100 in PBS, blocked with 10% goat serum (Beyotime), and incubated with primary antibodies F4/80 (ab6640, Abcam) and iNOS (ab178945, Abcam) overnight. After that, the cells underwent 1-h incubation with Alexa Fluor® 488-conjugated goat anti-rat antibody (ab150165, Abcam) or Alexa Fluor® 647-conjugated goat anti-rabbit antibody (ab150079, Abcam). Finally, the cells were incubated with 4′,6-diamidino-2-phenylindole (DAPI), followed by 3 washes with PBS.

### 2.4. Flow Cytometry

BMMs (100 *μ*L) were added with 5 *μ*L fluorescein isothiocyanate-labeled Annexin V and 10 *μ*L propidium iodide (BD Biosciences, San Jose, CA, USA) for 15 min away from light at room temperature. After that, 400 *μ*L binding buffer was added for flow cytometry analysis. The excitation wavelength was 488 nm, and 1 × 10^4^ cells were counted. All data were collected and processed by the CellQuest Pro software (BD Biosciences).

### 2.5. Extraction and Identification of EVs

When growing to about 80% confluence, the M1-BMMs were cultured in the EV-free special medium. After 48 h, the supernatant was collected and centrifuged (2500 g, 4°C, 15 min) to remove the scattered cells, followed by 30 min centrifugation (10000 g, 4°C) to remove the cell debris. After filtration using a 0.22 *μ*m filter (Merck Millipore, Burlington, MA, USA), the supernatant was centrifuged (20000 g, 30 min). Next, the supernatant was collected and centrifuged (10000 g, 70 min). The precipitate was collected, immersed in PBS, and centrifuged (10000 g, 70 min). The final obtained particles were suspended in PBS. The total protein content of EVs was measured using a bicinchoninic acid (BCA) kit (Beyotime). The morphology of EVs was observed under a transmission electron microscope (TEM, Hitachi). The size of EVs was analyzed by nanoparticle tracking analysis (NTA; Malvern Instruments, Ltd., Malvern, UK). The surface markers CD63, CD9, and calnexin were analyzed by Western blot analysis (WB). In addition, GW4869 (20 *μ*g/mL; Sigma-Aldrich) was added to M1-BMMs, and then the particles extracted as above were suspended in PBS as the GW group. RNase I (Thermo Fisher Scientific Inc., Waltham, MA, USA) was added to the obtained EVs and then heat-inactivated, which served as the RNase group.

### 2.6. Establishment and Treatment of MI Mouse Models

C57BL/6J mice (aged 6-8 weeks, 25-30 g) were used to establish the MI mouse model. The mice were anesthetized by an intraperitoneal injection of 3% pentobarbital sodium (80 mg/kg, Sigma-Aldrich). After the left chest was opened, the heart was exposed in the fourth and fifth intercostal spaces. The left anterior descending (LAD) coronary artery running in the same direction as the great cardiac vein was ligated with a 6-0 suture. After LAD ligation, the corresponding blood supply area turned white, and the electrocardiogram showed the manifestations of MI, such as elevated ST segment. The subsequent experiments were performed 30 min after ligation. Mice were allocated into the sham group (mice received thoracotomy without LAD ligation, MI group (mice received LAD ligation), GW group (mice were injected with 50 *μ*g/mL M1-BMM-conditioned medium containing GW4869 into MI surrounding area at multiple sites), and EVs group (mice were injected with 50 *μ*g/mL EVs into MI surrounding area at multiple sites), with 18 mice in each group. All mice were examined by echocardiography. After that, 6 mice were used for 2,3,5-triphenyltetrazolium chloride (TTC) staining, 6 mice were used for WB detection, and the others were used for hematoxylin and eosin (HE) staining.

### 2.7. Echocardiography

On the 7th and 28th days after EV injection, the cardiac function of mice was detected using the Vevo 2100 ultrasound imaging system for small animal imaging (Visual Sonics, Toronto, Canada). The mice were anesthetized with 3.5% isoflurane (RWD Life Science Co., Ltd., Shenzhen, China). After consciousness loss, the mice were further anesthetized with 2% isoflurane for echocardiography. The mice were fixed on a plate with constant temperature in the supine position. The body surface temperature, electrocardiogram, and respiratory curve of mice were recorded synchronously using a metal electrode. According to the left ventricular outflow tract, the papillary muscle was found by echocardiography. Next, the M-mode echocardiography was collected at the papillary muscle section, and the left ventricular end-diastolic diameter (LVEDD) and left ventricular end-systolic diameter (LVESD) were measured.

### 2.8. TTC Staining

The mice were euthanized immediately after echocardiography. The myocardium was collected and cut into 5 sections (1 mm) from the apex to the base. The infarct size (INF)/area-at-risk (AAR) was used to reflect the level of dead myocardium. The sections were incubated (15 min, 37°C) with PBS solution with 1% TTC and fixed in 10% formalin for 2 h. The ImageJ software was used to analyze the imaging. The INF/AAR was calculated using a weight-based method.

### 2.9. Masson's Trichrome Staining

The myocardial tissues were embedded in paraffin, sectioned (6 *μ*m) at an equal distance, and stained with Masson's trichrome (Beijing Solarbio Science & Technology Co., Ltd., Beijing, China). The staining and morphological changes were observed using an optical microscope (Olympus). The interstitial collagen deposition rate was measured by Masson's trichrome staining. The INF was quantitatively analyzed using the IPP 6.0 software.

### 2.10. HE Staining

The myocardial sections (6 *μ*m) were subjected to HE staining. In short, the sections were dewaxed using the biofilm transparency agent (Solarbio) and hydrated with gradient ethanol and double-distilled water. Afterward, the sections were stained with hematoxylin (Beyotime) for 5 min, washed with double-distilled water, and differentiated with 1% hydrochloric alcohol for 30 s. After washes with double-distilled water, the sections were stained with eosin (Beyotime) for 3 min, successively immersed in gradient ethanol and absolute ethanol, and then dehydrated and cleared in biofilm transparency agent. Finally, the sections were sealed and observed under a microscope (Olympus).

### 2.11. Terminal Deoxynucleotidyl Transferase- (TdT-) Mediated dUTP Nick-End Labeling (TUNEL) Staining

The myocardial apoptosis was detected by TUNEL staining using an *in situ* cell death detection kit (Roche Applied Science, Mannheim GmbH, Penzberg, Germany). Briefly, TUNEL and DAPI were used to stain the nuclei of apoptotic cardiomyocytes and the nuclei of all cardiomyocytes, respectively. The apoptotic rate = the number of apoptotic cardiomyocytes with positive staining/the total number of cardiomyocytes × 100%.

### 2.12. Immunohistochemistry

The myocardial sections (6 *μ*m) were incubated with the primary antibody CD31 (ab182981, Abcam) at 4°C overnight, and then with the secondary antibody (ab6721, Abcam). Next, 2,4-diaminobutyric acid complex (ZSGB-Bio Co., Ltd., Beijing, China) was added for color development. The nuclei were counterstained with 15% hematoxylin (Beyotime) and observed under the microscope (CKX41, Olympus).

### 2.13. Culture and Modeling of Myocardial Microvascular Endothelial Cells (MMECs)

The MMECs were extracted from C57BL/6J mice. The mouse heart was excised in ice-cold PBS. The atrium and right ventricle were removed, and the anterior wall of the left ventricle was preserved. After PBS washing for blood removal, the endocardium and epicardium were removed. About 1 × 1 × 1 mm slices were cut from the remaining tissues, which were then placed in a culture dish premoistened with FBS for 6 h. Next, 5 mL complete Dulbecco's modified Eagle's medium (DMEM) containing 10% FBS (Thermo Fisher) was added. After 48 h, the tissues were removed, and the culture medium was refreshed. The cells showed a cobblestone mosaic-like arrangement under the microscope (Olympus). VIII (ab275376, Abcam) was identified using the streptavidin-biotin complex (SABC) method. The cells showing brown cytoplasm with positive reaction were MMECs.

MMECs were cultured in glucose-free DMEM (#11966025, Thermo Fisher) in an anaerobic chamber (1% O_2_, 5% CO_2_, 37°C) to induce ischemic injury. After oxygen-glucose deprivation (OGD) treatment for 6 h, MMECs were treated with EVs [30, 31]. MMECs were allocated into the control group (no treatment), OGD group (treated with OGD), GW group (OGD-induced MMECs were treated with 50 *μ*g/mL M1-BMMs conditioned medium containing GW4869), and EVs group (OGD-induced MMECs were treated with 50 *μ*g/mL EVs). The analysis was performed after 24 h.

### 2.14. Internalization of Dil-Labeled EVs by MMECs

After NTA quantification, the extracted EVs were diluted in PBS and adjusted to the same number of particles. The EVs were mixed with Dil-dyeing working solution (Guangzhou RiboBio Co., Ltd., Guangzhou, Guangdong, China) at a volume ratio of 500 : 1. After incubation (37°C, 15 min) away from light, the EVs were centrifuged (16000 g, 60 min) with the supernatant discarded and washed 3 times with PBS to remove the excess dye. The labeled EVs were coincubated with MMECs for 48 h, followed by 3 washes with PBS and 15 min fixing in immunostaining fixative solution (Beyotime) at room temperature. After the addition of DAPI and 0.5 mL fluorescence mounting medium (Beyotime), the images were observed and collected under a fluorescence microscope (Olympus).

### 2.15. Cell Transfection

The MALAT1 siRNA and its si-negative control (NC) were provided by Shanghai GenePharma Co., Ltd. (Shanghai, China). As per the manufacturer's instructions, the siRNA and siNC were transfected into M1-BMMs using Lipofectamine 2000 (Invitrogen, Carlsbad, CA, USA) at a final concentration of 50 nM. The transfection efficiency was determined. After 48 h, the EVs were isolated and treated on OGD-induced MMECs (EVs-siNC/siMALAT1). After 24 h, the subsequent experiments were performed.

MicroRNA- (miR-) 25-3p mimic and its NC supplied by GenePharma were transfected into OGD-induced MMECs at a final concentration of 50 nM according to the manufacturer's instructions using Lipofectamine 2000 (Invitrogen). The OGD-induced MMECs were subjected to the combined treatment of EVs and miR-25-3p mimic/NC and grouped into EVs + mimic/NC. After 24 h, the subsequent experiments were performed.

### 2.16. 3-(4,5-Dimethylthiazol-2-yl)-2,5-Diphenyltetrazolium Bromide (MTT) Assay

The MMECs in each group were incubated with 10 *μ*L MTT solution (Beyotime) for 4 h. Next, 100 *μ*L dimethyl sulfoxide was added to each well. Finally, the optical density (OD) at 490 nm was recorded using a microplate reader (Bio-Rad, Hercules, CA, USA). The OD value was used to normalize the relative cell survival rate of the control group.

### 2.17. 5-Ethynyl-2′-Deoxyuridine (EdU) Assay

After trypsin treatment, the MMECs were seeded into 96-well plates. According to the manufacturer's instructions, the cell proliferation was assessed using an EdU proliferation detection (imaging) kit (C10310-3, RiboBio). Each well was added with 500 *μ*L diluted reagent A for 2 h incubation, washed 3 times with PBS, and then added with 500 *μ*L immunostaining fixative solution (Beyotime) for 30 min. Afterward, the fixative solution was discarded, and 500 *μ*L glycine was added for 5 min incubation. After PBS washing, 500 *μ*L immunostaining penetrant solution (Beyotime) was added, followed by PBS washing. Next, the MMECs were incubated with 500 *μ*L Apollo staining solution for 30 min. After the staining solution removal, the penetrant solution was added, followed by the addition of the Hoechst 33342 staining solution. Finally, the staining results were observed under the fluorescence microscope.

### 2.18. Angiogenesis Assay

The 12-well cell culture plates coated with 500 *μ*L cold Matrigel (4°C) functioned as the basis of angiogenesis. The gel was placed in an incubator (5% CO_2_, 37°C) for 30 min. MMECs (6 × 10^4^ cells/well) in each group were seeded onto the gel for incubation (5% CO_2_, 37°C). After 12 h, blood vessel formation was observed and recorded using a microscope and analyzed using the ImageJ software (NIH, Bethesda, MD, USA).

### 2.19. RNA-Fluorescence *In Situ* Hybridization (FISH)

FAM-labeled oligo DNA probe of MALAT1 was obtained from GenePharma. The MMECs were cultured in 24-well plates (1 × 10^5^ cells). After 24 h of incubation, the medium was discarded. The MMECs were washed 3 times with PBS and fixed in polyoxymethylene and prehybridized (1 PBS/0.5% Triton X-100). Next, the MMECs were hybridized at 42°C overnight with MALAT1 probe in the hybridization buffer. Following washes with the hybridization buffer and washing buffer, DAPI (Beyotime) was used for nuclear staining. Finally, the Leica SP5 confocal microscope (Leica Microsystems, Wetzlar, Germany) was used to observe and photograph the MMECs.

### 2.20. Nuclear and Cytoplasmic Fractionation Assay

The extract was prepared using the NE-PER nuclear and cytoplasmic extraction kit (Thermo Fisher) in strict accordance with the manufacturer's instructions. In short, the MMEC precipitate was suspended in cytoplasmic extraction reagent I containing 1 mL phenylmethylsulfonyl fluoride (PMSF). After 10 min incubation on ice, the second cytoplasmic extraction reagent II was added for 1 min incubation on ice, followed by centrifugation (16000 g, 5 min). The supernatant was put into the precooled tube, which was the cytoplasmic extract. The insoluble precipitate containing coarse nuclei was resuspended in the nuclear extraction reagent (NER) containing 1 mL PMSF (PMSF : NER = 1 : 100) by a 15 s vortex oscillation every 10 min 4 times. After 15 s incubation on ice and centrifugation (16000 g, 10 min), the obtained supernatant was the nuclear extract. Finally, MALAT1 expression in nuclear and cytoplasmic extracts was analyzed using reverse transcription quantitative polymerase chain reaction (RT-qPCR).

### 2.21. Bioinformatics Analysis

Through the LncDisease database (http://www.cuilab.cn/lncrnadisease) [32], MI-related lncRNAs were retrieved. The downstream miRs of MALAT1 were predicted through the LncACT database (http://www.bio-bigdata.net/LncACTdb/index.html?quick=SLCO4A1-AS1) [33], the RAID database (http://www.rna-society.org/raid2/index.html) [34], the RNA22 database (https://cm.jefferson.edu/rna22/) [35], and the starBase database (http://starbase.sysu.edu.cn/index.php) [36]. Through the GEO database (https://www.ncbi.nlm.nih.gov/geo/), the MI microarray GSE97320 (including three normal samples and three disease samples) was obtained. Taking the normal samples as the control, the limma package of R language was used for differential analysis, with ∣logFC | >1 and *p* value < 0.05 as the screening criteria. Through the TargetScan database (http://www.targetscan.org/vert_71/) [37], the downstream target gene of miR-25-3p was predicted.

### 2.22. RNA Immunoprecipitation (RIP)

Anti-AGO2 RIP was performed in 293T cells (American Type Culture Collection (ATCC), Manassas, Virginia, USA)) transfected with miR-25-3p mimic or NC. In short, the 293T cell lysis buffer was preblocked by protein G beads (Invitrogen) and then incubated (4°C, 90 min) with Anti-AGO G beads (Pierce Biotechnology, Waltham, MA, USA). After centrifugation (600 g, 1 min), the beads were collected, washed 5 times with radio-immunoprecipitation assay (RIPA) buffer, and resuspended in Tris-HCl (50 mmol/L, pH = 7). The beads were incubated (70°C, 45 min) for reverse cross-linking. Next, the RNA was extracted for quantification by RT-qPCR.

### 2.23. Dual-Luciferase Reporter Gene Assay

The binding sites of miR-25-3p with MALAT1 and cell division control protein 42 (CDC42) were predicted through the RNA22 database (https://cm.jefferson.edu/rna22/) [35] and the starBase database (http://starbase.sysu.edu.cn/index.php) [36]. The dual-luciferase reporter gene assay was used to verify their binding relationships. The 3′UTR sequences (containing binding sites with miR-25-3p) of MALAT1 and CDC42 wild type (WT) were inserted into the pmirGLO vector (Promega Corp., Madison, WI, USA) to prepare the corresponding WT luciferase reporter genes MALAT1-WT and CDC42-WT. After the mutation in the complementary 3′UTR sites of miR-25-3p in MALAT1 and CDC42, the mutant (MUT) reporter genes MALAT1-MUT and CDC42-MUT were constructed. Next, the above reporter genes were cotransfected with miR-25-3p mimic or mimic NC into 293T cells (ATCC), respectively. Finally, the dual-luciferase reporter gene detection system (Promega) was used to detect the luciferase activity in cell lysates.

### 2.24. WB

Total protein was extracted from mouse myocardial tissues or MMECs using RIPA lysis buffer (Beyotime) and protease inhibitor cocktail (Roche Molecular Biochemicals, Basel, Switzerland). The protein concentration was measured using a BCA protein kit (Beyotime). The protein was separated in 12% sodium dodecyl sulfate-polyacrylamide gel electrophoresis and then transferred to pure nitrocellulose membranes (Pall Life Science, Ann Arbor, MI, USA). Next, the membranes were incubated overnight at 4°C with primary antibodies CD63 (1 : 1000, ab217345), CD9 (1 : 2000, ab92726), calnexin (1 *μ*g/mL, ab22595), vascular endothelial growth factor (VEGF) (1 : 1000, ab32152), CDC42 (1 : 10000, ab187643), p-MEK (1 : 1000, ab96379), p-ERK (1 : 500, ab131438), and GAPDH (1: 10000, ab181602). After 3 washes with PBS containing Triton X-100, the membranes were incubated with the horseradish peroxidase-labeled goat anti-rabbit secondary antibody (1 : 2000, ab205718) for 2 h. The enhanced chemiluminescence (Merck Millipore) was used for development. The ImageJ v1.48u software (NIH) was used for analysis. All antibodies were purchased from Abcam.

### 2.25. RT-qPCR

To detect MALAT1 expression, total RNA was extracted from mouse myocardial tissues or MMECs using RNAzol reagent (Sigma-Aldrich). The SuperScript III reverse transcriptase (Thermo Fisher) was used for reverse transcription, and the Power™ SYBR™ green master mix (Applied Biosystems, Inc., Carlsbad, CA, USA) was used to prepare all PCR reaction systems. To detect miR-25-3p expression, miRs were extracted using the miRNA isolation kit (RMI050, Geneaid Biotech Ltd., Taipei, Taiwan, China). miRNA reverse transcription was performed using the miScript II RT kit (QIAGEN, Valencia, CA, USA). The PCR reaction system was prepared using the miScript SYBR Green PCR kit (QIAGEN). All PCR reactions were performed using the CFX96 Touch Deep Well™ real-time PCR detection system (Bio-Rad). The primers are shown in Table 1.

### 2.26. Statistical Analysis

All data were processed using SPSS 21.0 (IBM Corp., Armonk, NY, USA). The measurement data were expressed as mean ± standard deviation. Comparison between two groups was analyzed using the *t*-test. Comparison among groups was analyzed using one-way analysis of variance (ANOVA), followed by Tukey's multiple comparisons test. *p* < 0.05 was indicative of a statistically significant difference.

## 3. Results

### 3.1. M1-BMMs-EVs Were Successfully Obtained

**Figure 1:**
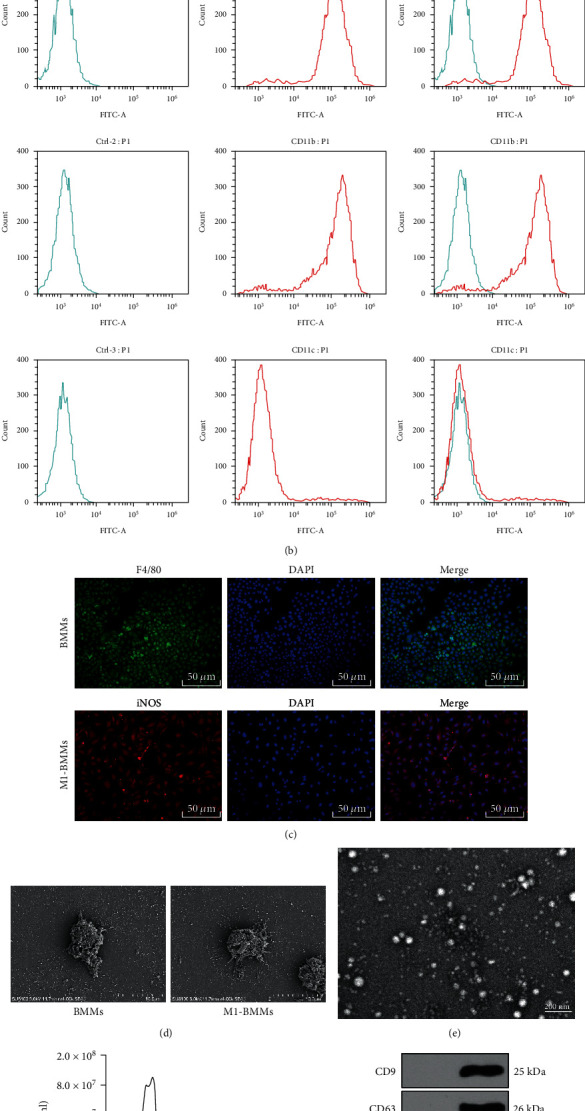
M1-BMMs-EVs are successfully obtained. (a) BMMs and M1-BMMs were observed under an inverted microscope. (b) BMMs were identified using flow cytometry. (c) BMMs and M1-BMMs were identified by immunofluorescence. (d) BMMs and M1-BMMs were observed under a scanning electron microscope. (e) M1-BMMs-EVs were observed under a TEM. (f) EV particle size was analyzed by NTA. (g) The surface markers of EVs were identified using WB with M1-BMM-conditioned medium containing GW4869 as control.

Macrophages, as the core regulatory cell population in inflammatory responses, play an important role in the process of ventricular remodeling after MI [14–16]. Previous studies have provided evidence that in the process of atherosclerosis, M1-BMMs can secrete EVs to promote the proliferation and migration of vascular smooth muscle cells, thereby aggravating vascular injury [24, 25]. To study the role of M1-BMMs-EVs in MI, we first extracted and identified mouse BMMs. Under the inverted microscope, we observed that the cells had typical macrophage characteristics with polygonal or spindle shapes and filamentous pseudopods and tentacles (Figure 1(a)). The results of flow cytometry for BMM markers showed that the cultured cells showed 92.19% F4/80 expression, 89.59% CD11b expression, and 4.94% CD11c expression (Figure 1(b)), which were consistent with the characteristics of macrophages. In addition, we found that more than 95% of the cells expressed F4/80 with green fluorescence (Figure 1(c)). As shown by the observations under the scanning electron microscope, the cells were flat and rod-shaped with a few tentacles (Figure 1(d)). All these results indicated that BMMs were successfully obtained. After stimulation using LPS and IFN-*γ*, the cells became flat and long rod-shaped with longer and thinner pseudopodia, which indicated that the cells polarized towards M1-BMMs (Figure 1(a)). Immunofluorescence results revealed that more than 90% of cells expressed iNOS with red fluorescence (Figure 1(c)). Additionally, many slender and long tentacles were observed on the cell membrane under a scanning electron microscope (Figure 1(d)). From all above, we confirmed that M1-BMMs were successfully obtained.

Next, M1-BMMs-EVs were extracted, which were teacup-shaped or hemispherical-shaped with one side concave inward EVs and membrane structure and a diameter of about 30-120 nm under a TEM (Figure 1(e)). The average size of EVs was 104.2 ± 3.5 nm (Figure 1(f)). As shown by WB results, EVs showed the expression of positive markers CD9 and CD63 with no expression of the negative marker calnexin (Figure 1(g)). Taken together, M1-BMMs-EVs were successfully obtained.

### 3.2. M1-BMMs-EVs Aggravated MI in Mice

**Figure 2:**
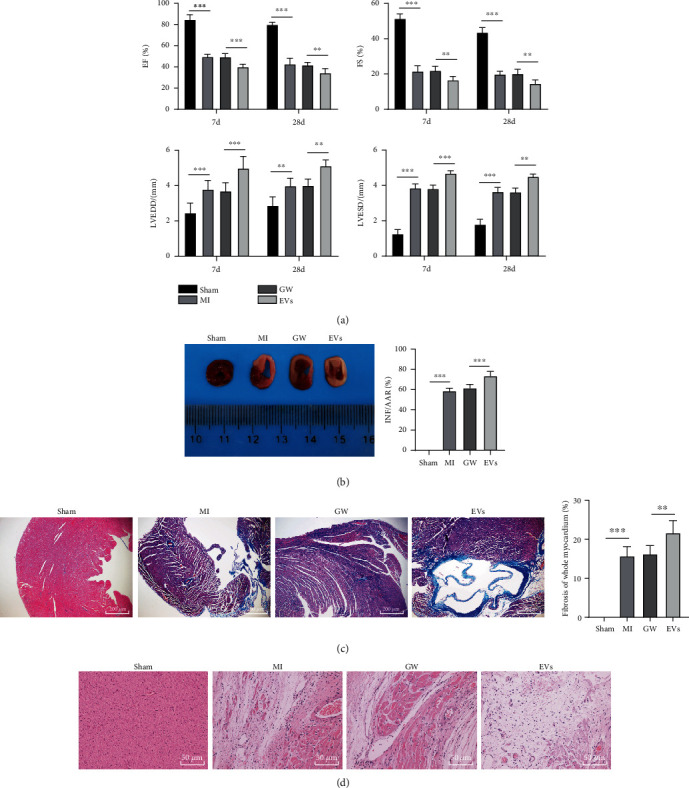
M1-BMMs-EVs aggravate MI in mice. The MI mouse model was established by LAD coronary artery ligation and treated with M1-BMMs-EVs, with the injection of M1-BMM-conditioned medium containing GW4869 as the control. (a) Cardiac function of mice in each group on the 7th and 28th days after injection was detected by echocardiography, *n* = 18. (b) TTC staining was used to detect the infarct area of mice in each group on the 28th day, *n* = 6. (c, d) Representative images of Masson's trichrome staining and HE staining for the detection of the fibrosis of mice in each group on the 28th day, in animal experiments, *n* = 6. *N* means the number of animals used in each group. The data were expressed as mean ± standard deviation. One-way ANOVA was used to analyze comparisons among multiple groups followed by Tukey's multiple comparisons test. ^∗∗^*p* < 0.01, ^∗∗∗^*p* < 0.001.

To verify the role of M1-BMMs-EVs in MI, we first established the MI mouse model by the LAD ligation. We found that the cardiac function of MI mice was damaged (all *p* < 0.001) (Figure 2(a)), which indicated the successful establishment of the MI model. Next, we injected M1-BMMs-EVs into the heart of MI mice at multiple sites *in situ* with the injection of an M1-BMM-conditioned medium containing GW4869 as the control. Echocardiography was performed on the 7th and 28th days after the injection. It was found that M1-BMMs-EV treatment aggravated the cardiac dysfunction of MI mice (all *p* < 0.01) (Figure 2(a)). The mice were euthanized on the 28th day, and the myocardial tissue section staining results suggested that EV-treated mice showed larger infarct areas and more serious fibrosis (all *p* < 0.01) (Figures 2(b)–2(d)). From all above, M1-BMMs-EVs aggravated MI in mice.

### 3.3. M1-BMMs-EVs Inhibited Angiogenesis and Myocardial Regeneration after MI in Mice

**Figure 3:**
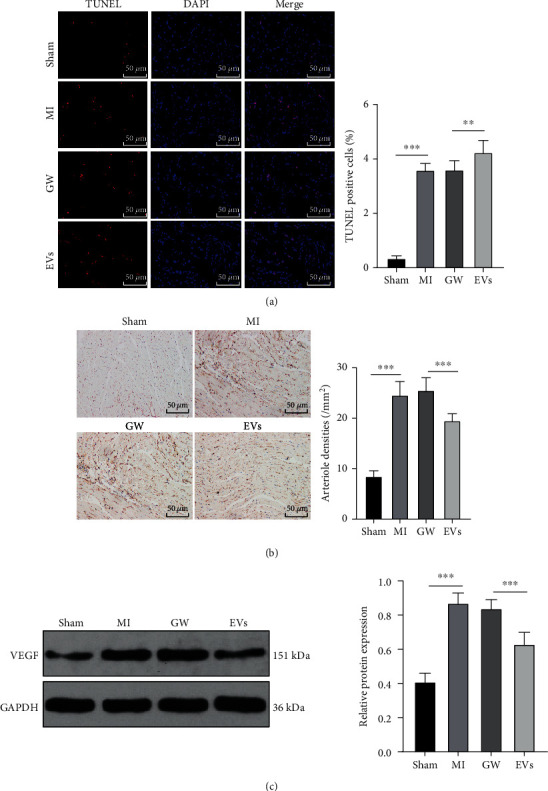
M1-BMMs-EV treatment inhibits angiogenesis and myocardial regeneration after MI in mice. The MI mice were treated with M1-BMMs-EVs with the injection of an M1-BMM-conditioned medium containing GW4869 as the control. The myocardial tissues on the 28th day were taken. (a) The cell apoptosis of mice in each group was detected using TUNEL staining. (b) The expression of CD31 was detected by immunohistochemistry. (c) The expression of VEGF was detected by WB. In animal experiments, *n* = 6. The data were expressed as mean ± standard deviation. One-way ANOVA was used to analyze comparisons among multiple groups followed by Tukey's multiple comparisons test. ^∗∗^*p* < 0.01, ^∗∗∗^*p* < 0.001.

Numerous studies have shown that angiogenesis and myocardial regeneration are crucial for cardiac function following MI [38–40]. As shown by TUNEL staining results, apoptotic cells were notably increased in the infarct area of MI mice, and compared with the GW group, apoptotic cells were further increased in the infarct area of MI mice in the EVs group (all *p* < 0.01) (Figure 3(a)). On the other hand, CD31 and VEGF are markers of angiogenesis [41, 42]. According to our results, EV treatment reduced CD31 and VEGF expressions (all *p* < 0.001) (Figures 3(b) and 3(c)). Briefly, M1-BMMs-EV treatment inhibited angiogenesis and myocardial regeneration after MI in mice.

### 3.4. M1-BMMs-EVs Inhibited Angiogenesis and Myocardial Regeneration in OGD-Treated MMECs

**Figure 4:**
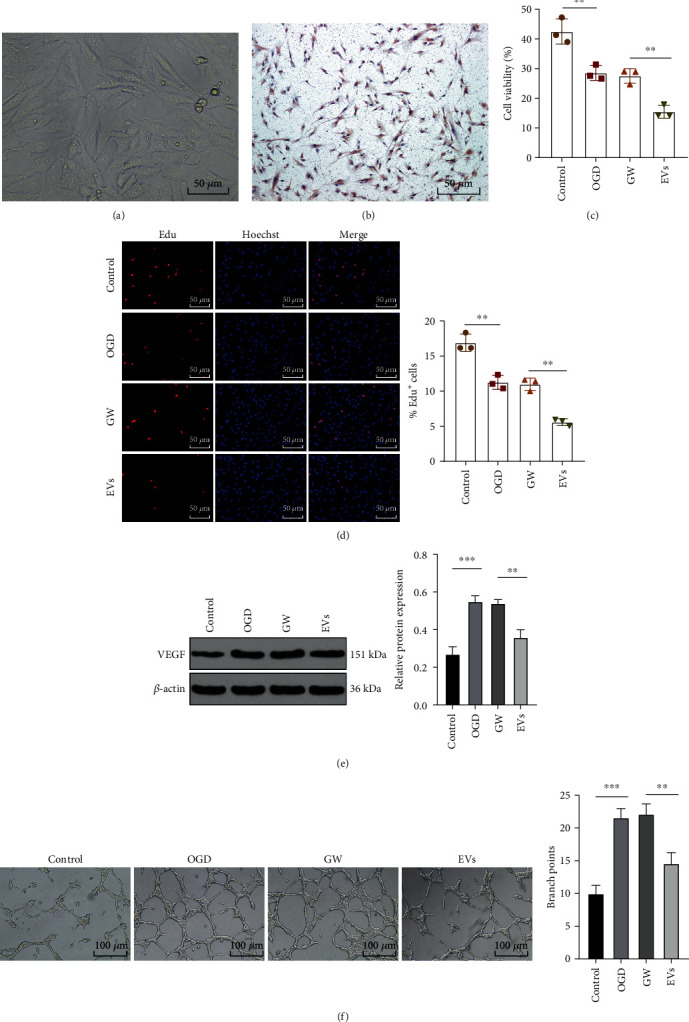
M1-BMMs-EVs inhibit angiogenesis and myocardial regeneration in OGD-treated MMECs. MMECs were treated with OGD to simulate myocardial ischemia and then treated with M1-BMMs-EVs, with the treatment of an M1-BMM-conditioned medium containing GW4869 as the control. (a) The morphology of MMECs was observed under a microscope. (b) VIII was detected by the SABC method to identify MMECs. (c) The cell viability was detected using the MTT assay. (d) The EdU assay was used to detect cell proliferation. (e) VEGF expression in MMECs was detected using WB. (f) Blood vessel formation was detected by angiogenesis assay. The cell experiment was repeated three times, and the data were expressed as mean ± standard deviation. One-way ANOVA was used to analyze comparisons among multiple groups followed by Tukey's multiple comparisons test. ^∗∗^*p* < 0.01, ^∗∗∗^*p* < 0.001.

Through the above *in vivo* experiments, we identified the effect of M1-BMMs-EVs on MI, which was then further verified by *in vitro* experiments. MMECs were extracted and observed to be cobblestone mosaic-like arrangement with positive VIII under a microscope (Figures 4(a) and 4(b)). MMECs were treated with OGD to simulate myocardial ischemia and then treated with M1-BMMs-EVs. According to our results, EV treatment remarkably reduced the viability (both *p* < 0.01) (Figure 4(c)) and inhibited the proliferation of OGD-treated MMECs (both *p* < 0.001) (Figure 4(d)). In addition, after EV treatment, VEGF expression in OGD-treated MMECs was inhibited (both *p* < 0.01) (Figure 4(e)), and angiogenesis was reduced (both *p* < 0.01) (Figure 4(f)). These results suggested that M1-BMMs-EVs inhibited angiogenesis and myocardial regeneration in OGD-treated MMECs.

### 3.5. M1-BMMs-EVs Carried lncRNA MALAT1 into MMECs

**Figure 5:**
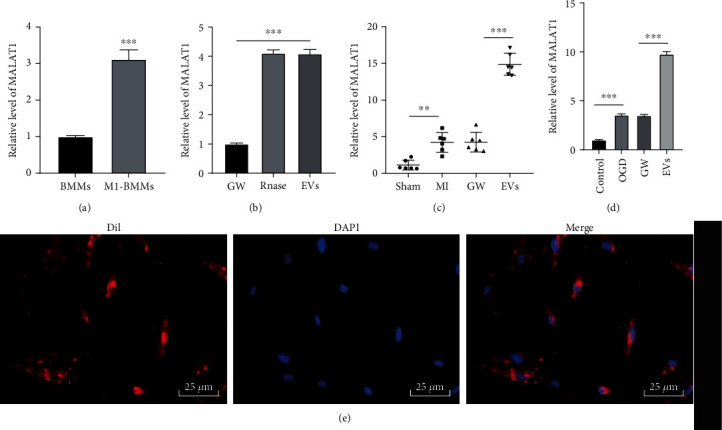
M1-BMMs-EVs carry lncRNA MALAT1 into MMECs. (a, b) RT-qPCR was used to detect the expression of MALAT1 in M1-BMMs and M1-BMMs-EVs. (c) RT-qPCR was used to detect the expression of MALAT1 in myocardial tissues of mice in each group. (d) RT-qPCR was used to detect the expression of MALAT1 in MMECs in each group. (e) Dil-labeled EVs coincubated with MMECs were detected using immunofluorescence, in animal experiments, *n* = 6. The cell experiment was repeated three times, and the data were expressed as mean ± standard deviation. The data between two groups were analyzed using the independent sample *t*-test, and the data among multiple groups were compared using one-way ANOVA followed by Tukey's multiple comparisons test. ^∗∗^*p* < 0.01, ^∗∗∗^*p* < 0.001.

Through the LncDisease database (http://www.cuilab.cn/lncrnadisease) [32], MI-related lncRNAs were retrieved. Among them, lncRNA MALAT1 plays an important role in cardiac diseases [43, 44]. Moreover, it has been proposed that EVs can carry MALAT1 [45]. Therefore, MALAT1 expression was detected. We found that MALAT1 was highly expressed in M1-BMMs (*p* < 0.001) (Figure 5(a)). MALAT1 also showed an abnormal upregulation in M1-BMMs-EVs with no obvious difference between the EVs group and RNase group (*p* < 0.001) (Figure 5(b)), which indicated that MALAT1 was encapsulated in EVs. In addition, compared with that in MI mice and OGD-treated MMECs with GW4869 treatment, MALAT1 expression was notably increased in EV-treated mice and MMECs (all *p* < 0.01) (Figures 5(c) and 5(d)). Under a fluorescence microscope, it was found that Dil-labeled M1-BMMs-EVs were internalized by MMECs (Figure 5(e)). Taken together, after internalization by MMECs, M1-BMMs-EVs released MALAT1 into MMECs to upregulate MALAT1 expression in MMECs.

### 3.6. MALAT1 Knockdown Attenuated the Inhibition of M1-BMMs-EVs on Angiogenesis and Myocardial Regeneration

**Figure 6:**
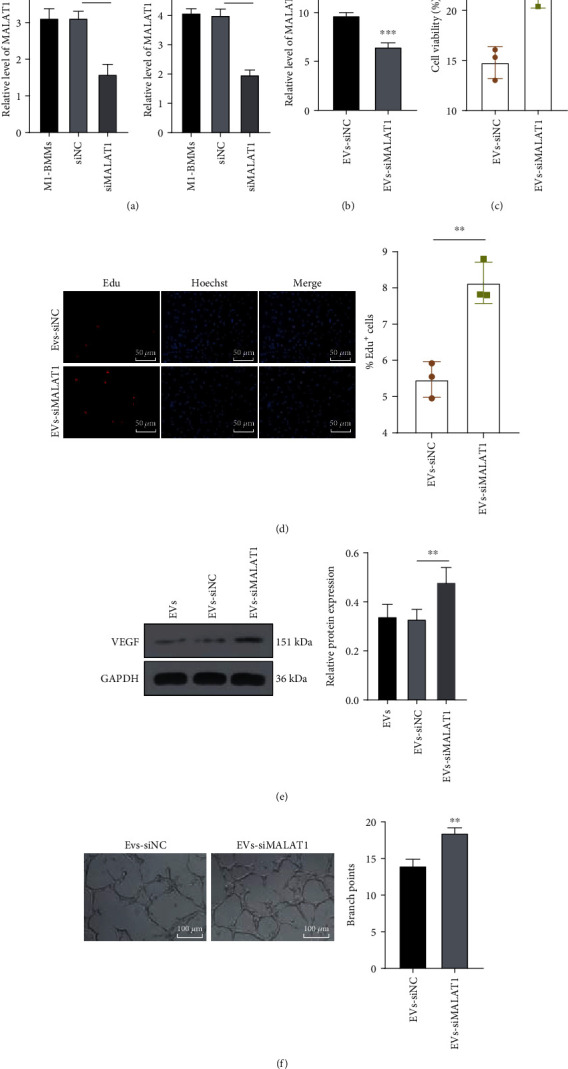
MALAT1 knockdown attenuates the inhibition of M1-BMMs-EVs on angiogenesis and myocardial regeneration. M1-BMMs were treated with siRNA of MALAT1 (siMALAT1) with the transfection of siNC as the control, and then EVs were extracted to treat OGD-induced MMECs. (a, b) The effects of siMALAT1 on the expression of MALAT1 in M1-BMMs, EVs, and OGD-induced MMECs were detected by RT-qPCR. (c) The cell viability was detected by MTT assay. (d) The cell proliferation was detected by EdU assay. (e) VEGF expression in MMECs was detected using WB. (f) Blood vessel formation was detected by angiogenesis assay. The cell experiment was repeated three times, and the data were expressed as mean ± standard deviation. The data between two groups in (b–d) and (f) were analyzed using the independent sample *t*-test, and the data among multiple groups in (a) and (e) were compared using one-way ANOVA followed by Tukey's multiple comparisons test. ^∗∗^*p* < 0.01, ^∗∗∗^*p* < 0.001.

Subsequently, we downregulated MALAT1 expression by transfecting si-MALAT1 into M1-BMMs with siNC as the control to verify the role of M1-BMMs-EVs-carried MALAT1 in angiogenesis and myocardial regeneration (*p* < 0.001) (Figure 6(a)). The EVs were then extracted. We found that siMALAT1 transfection decreased MALAT1 expression in EVs (*p* < 0.001) (Figure 6(a)). Next, OGD-treated MMECs were treated with the above EVs (EVs-siMALAT1/siNC). It was observed that MALAT1 expression was also remarkably reduced in MMECs after siMALAT1 treatment (*p* < 0.001) (Figure 6(b)). Besides, after MALAT1 knockdown in M1-BMMs, M1-BMMs-EVs-treated OGD-induced MMECs promoted cell proliferation (both *p* < 0.01) (Figures 6(c) and 6(d)) and partially increased cell angiogenesis (both *p* < 0.01) (Figures 6(e) and 6(f)). These results revealed that MALAT1 knockdown attenuated the inhibition of M1-BMMs-EVs on angiogenesis and myocardial regeneration.

### 3.7. MALAT1 Competitively Bound to miR-25-3p to Promote CDC42 Expression

**Figure 7:**
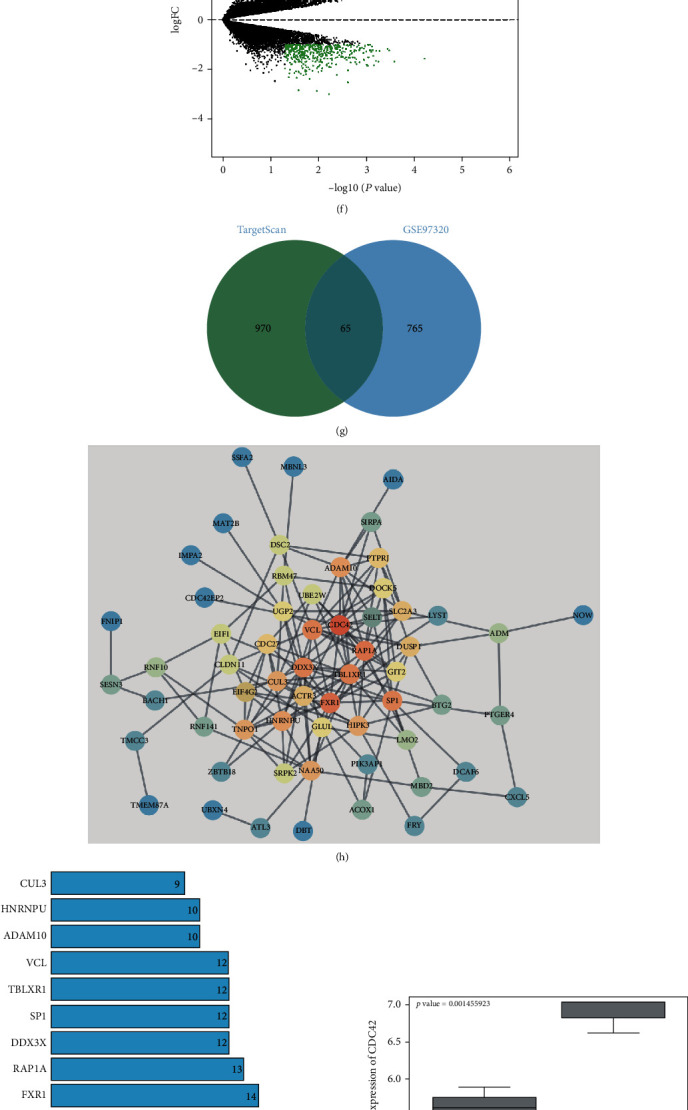
MALAT1 competitively binds to miR-25-3p to promote CDC42 expression. (a) RNA22 database (https://cm.jefferson.edu/rna22/) was used to analyze the localization of MALAT1 in the cytoplasm. (b) RNA-FISH assay was used to detect the fluorescence localization of lncRNA MALAT1 in MMECs. (c) RT-qPCR was used to detect MALAT1 expression after nuclear and cytoplasmic fractionation assay. (d) MALAT1 downstream miRs were predicted; four circles in the figure showed the prediction results of four databases, and the middle part showed the intersection of four databases. (e) RT-qPCR was used to analyze the expression of 6 candidate miRs in MI mice and OGD-treated cells. (f) The volcano map of the differentially expressed genes in microarray GSE97320; the abscissa showed -log10p value, and the ordinate represented logFC; each point in the figure represented a gene with the red points indicative of upregulated genes, and the green points indicative of downregulated genes in MI. (g) The miR-25-3p downstream target genes were predicted through the TargetScan database (http://www.targetscan.org/vert_71/); two circles in the figure represented the prediction results and the upregulated genes in microarray GSE97320, respectively, and the middle part represented the intersection. (h) The interaction analysis of miR-25-3p candidate target genes was performed; each circle in the figure represented a gene, and the line between circles indicated the interaction between genes; more interaction genes suggested a higher degree value of a gene and a more core position in the network. (i) Statistics of the top 10 genes with the highest core degree; the abscissa represented the degree value, and the ordinate represented the gene name. (j) CDC42 expression in microarray GSE97320; the abscissa represented the sample type, the ordinate represented the expression value, and the upper left was the differential *p* value. (k) RNA22 database (https://cm.jefferson.edu/rna22/) and starBase database (http://starbase.sysu.edu.cn/index.php) were used to analyze the binding sites of miR-25-3p with MALAT1 or CDC42. (l) Dual-luciferase reporter gene assay was used to analyze the binding relationship between miR-25-3p and MALAT1 or CDC42. (m) RIP assay was used to detect the binding relationship between miR-25-3p and MALAT1. (n) The expression of MALAT1 in MMECs was detected by RT-qPCR. (o) The expression of CDC42 was detected by WB. In animal experiments, *n* = 6. The cell experiment was repeated three times, and the data were expressed as mean ± standard deviation. The data between two groups in (c), (j), and (k) were analyzed using an independent sample *t*-test, and the data among multiple groups in (l) and (m) were analyzed using one-way ANOVA followed by Tukey's multiple comparisons test. ^∗∗^*p* < 0.01, ^∗∗∗^*p* < 0.001.

The above results have verified the role of MALAT1 in MI. We then studied the downstream molecular mechanism. Firstly, we predicted through the RNA22 database (https://cm.jefferson.edu/rna22/) that MALAT1 was localized in the cytoplasm (Figure 7(a)). Our RNA-FISH assay and nuclear and cytoplasmic fractionation assay confirmed that MALAT1 was mainly located in the cytoplasm of MMECs (both *p* < 0.001) (Figures 7(b) and 7(c)), which suggested that MALAT1 may play a role through the competitive endogenous RNA (ceRNA) mechanism. Therefore, we predicted the downstream miRs of MALAT1 through four databases (LncACT (http://www.bio-bigdata.net/LncACTdb/index.html?quick=SLCO4A1-AS1), RAID (http://www.rna-society.org/raid2/index.html), RNA22 (https://cm.jefferson.edu/rna22/), and starBase (http://starbase.sysu.edu.cn/index.php)). After intersecting the predicted results of databases, 6 candidate miRs were identified (Figure 7(d)). Among the 6 candidate miRs, miR-25-3p was identified to show the most significant differential expression in MI (all *p* < 0.001) (Figure 7(e)). In addition, a study has pointed out the involvement of miR-25-3p in the regulation of MI [30]. Thus, we selected miR-25-3p for further experiments.

Next, the differential analysis of MI microarray GSE97320 was carried out (Figure 7(f)). The miR-25-3p downstream target genes were predicted through the TargetScan database (http://www.targetscan.org/vert_71/), and after the intersection of prediction results with the upregulated genes in microarray GSE97320, 65 candidate genes were finally identified (Figure 7(g)). Furthermore, the interaction analysis of these 65 candidate genes was performed, and the gene interaction network was plotted (Figure 7(h)). After analysis of the degree value of the core genes in the gene interaction network (Figure 7(i)), we found that the CDC42 gene was the most core gene in the network. Additionally, microarray GSE97320 analysis showed that CDC42 was remarkably upregulated in MI (*p* < 0.01) (Figure 7(j)).

Subsequently, the binding sites between MALAT1 and miR-25-3p and between miR-25-3p and CDC42 were predicted (Figure 7(k)). RIP and dual-luciferase reporter gene assay verified the binding relationships between MALAT1 and miR-25-3p and between miR-25-3p and CDC42 (all *p* < 0.001) (Figures 7(l) and 7(m)). In addition, relative to MMECs in the EVs-siNC group, MMECs in the EVs-siMALAT1 group showed remarkably increased miR-25-3p expression and decreased CDC42 expression (all *p* < 0.01) (Figures 7(n) and 7(o)). From all above, we confirmed that M1-BMMs-EVs could carry MALAT1 into MMECs, which competitively bound to miR-25-3p to promote CDC42 expression.

### 3.8. miR-25-3p Overexpression Partially Reversed the Inhibitory Effects of EVs on Angiogenesis and Myocardial Regeneration

**Figure 8:**
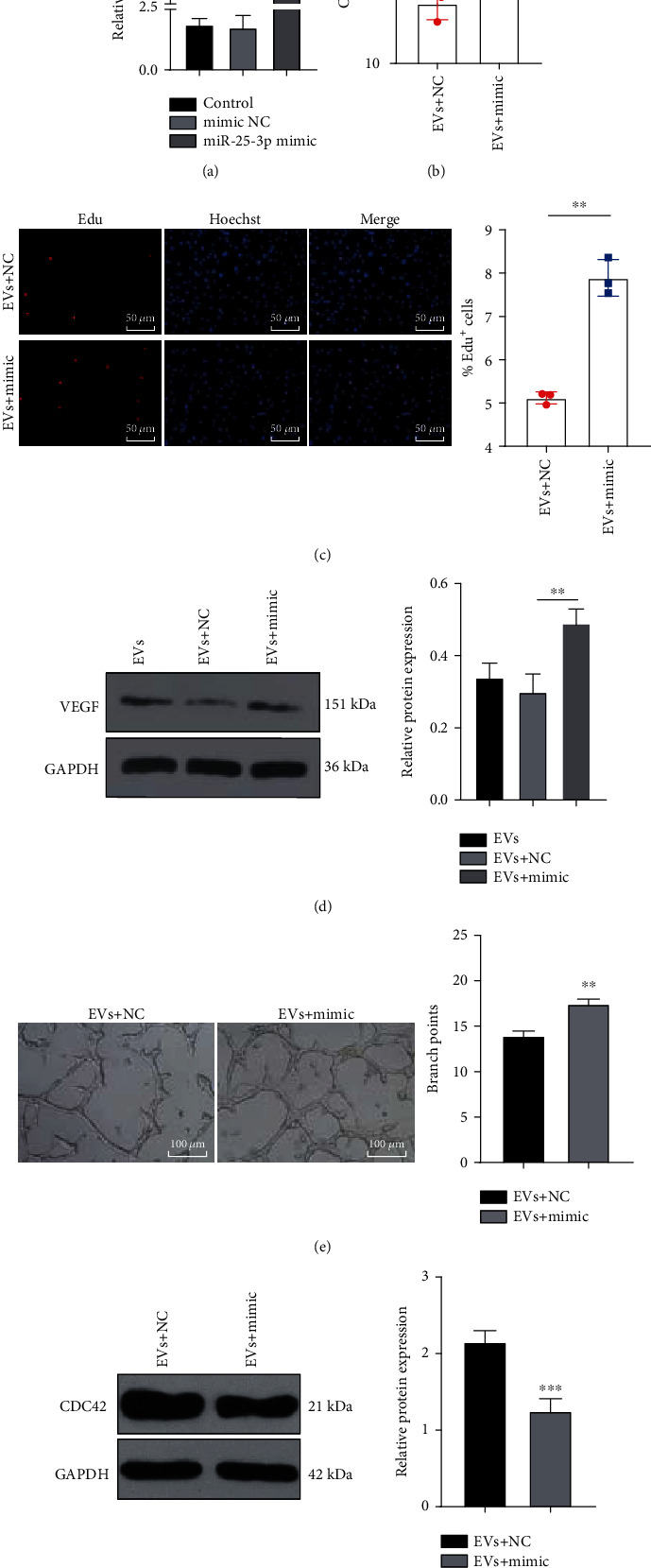
miR-25-3p overexpression partially reverses the inhibitory effects of EVs on angiogenesis and myocardial regeneration. OGD-induced MMECs were treated with EVs and miR-25-3p mimic/mimic NC. (a) The effects of miR-25-3p mimic on miR-25-3p expression were evaluated by RT-qPCR. (b) The cell viability was detected by MTT assay. (c) EdU assay was used to detect the cell viability. (d) VEGF expression in MMECs was detected using WB. (e) Blood vessel formation was detected by angiogenesis assay. (f) WB was used to detect the effect of miR-25-3p overexpression on the expression of CDC42. The cell experiment was repeated three times, and the data were expressed as mean ± standard deviation. The data between two groups in (b), (c), (e), and (f) were analyzed using the independent sample *t*-test, and the data among multiple groups in (a) and (d) were analyzed using one-way ANOVA followed by Tukey's multiple comparisons test. ^∗∗^*p* < 0.01, ^∗∗∗^*p* < 0.001.

To verify the role of miR-25-3p in MI, we treated OGD-induced MMECs with miR-25-3p mimic and EVs. The transfection efficiency of miR-25-3p mimic was verified by RT-qPCR (*p* < 0.001) (Figure 8(a)). The combined treatment of EVs and miR-25-3p mimic increased cell viability, promoted cell proliferation (both *p* < 0.01) (Figures 8(b) and 8(c)), and enhanced the angiogenesis ability of OGD-treated cells (both *p* < 0.01) (Figures 8(d) and 8(e)). In addition, compared with the EVs + NC group, the EVs + mimic group exhibited decreased CDC42 expression (*p* < 0.001) (Figure 8(f)). Overall, miR-25-3p overexpression partially annulled the inhibitory effects of EVs on angiogenesis and myocardial regeneration.

### 3.9. EV-Carried MALAT1 Activated the MEK/ERK Pathway

**Figure 9:**
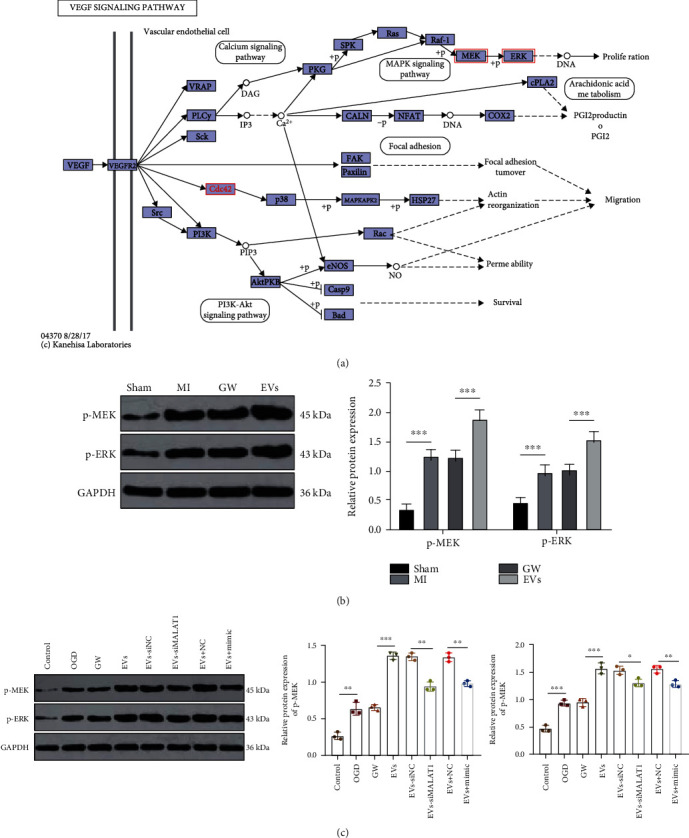
M1-BMMs-EV-carried MALAT1 activates the MEK/ERK pathway. (a) KEGG analysis (https://www.kegg.jp/kegg-bin/show_pathway?ko04370+K04393) for the downstream pathway of CDC42. (b, c) WB was used to detect the phosphorylation levels of MEK and ERK in MI mice and OGD-treated MMECs. The cell experiment was repeated three times, and the data were expressed as mean ± standard deviation. The data among multiple groups were analyzed using two-way ANOVA followed by Tukey's multiple comparisons test. ^∗∗^*p* < 0.01, ^∗∗∗^*p* < 0.001.

We have verified the ceRNA mechanism of MALAT1. To explore the downstream pathway of CDC42, we performed KEGG analysis (https://www.kegg.jp/kegg-bin/show_pathway?ko04370+K04393). It was found that the MEK/ERK pathway downstream of CDC42 was involved in proliferation (Figure 9(a)). The role of the MEK/ERK pathway in myocardial ischemia-reperfusion has also been reported [46]. Hence, we speculated that the MEK/ERK pathway might play a role in the effects of EVs on MI. According to our results, MEK and ERK were highly phosphorylated in MI mice and OGD-treated MMECs. EV treatment further promoted the phosphorylation of MEK and ERK (all *p* < 0.001) (Figures 9(b) and 9(c)). Moreover, the phosphorylation of MEK and ERK was inhibited after MALAT1 knockdown or miR-25-3p overexpression (all *p* < 0.05) (Figure 9(c)). These results verified that M1-BMMs-EV-carried MALAT1 activated the MEK/ERK pathway.

## 4. Discussion

**Figure 10:**
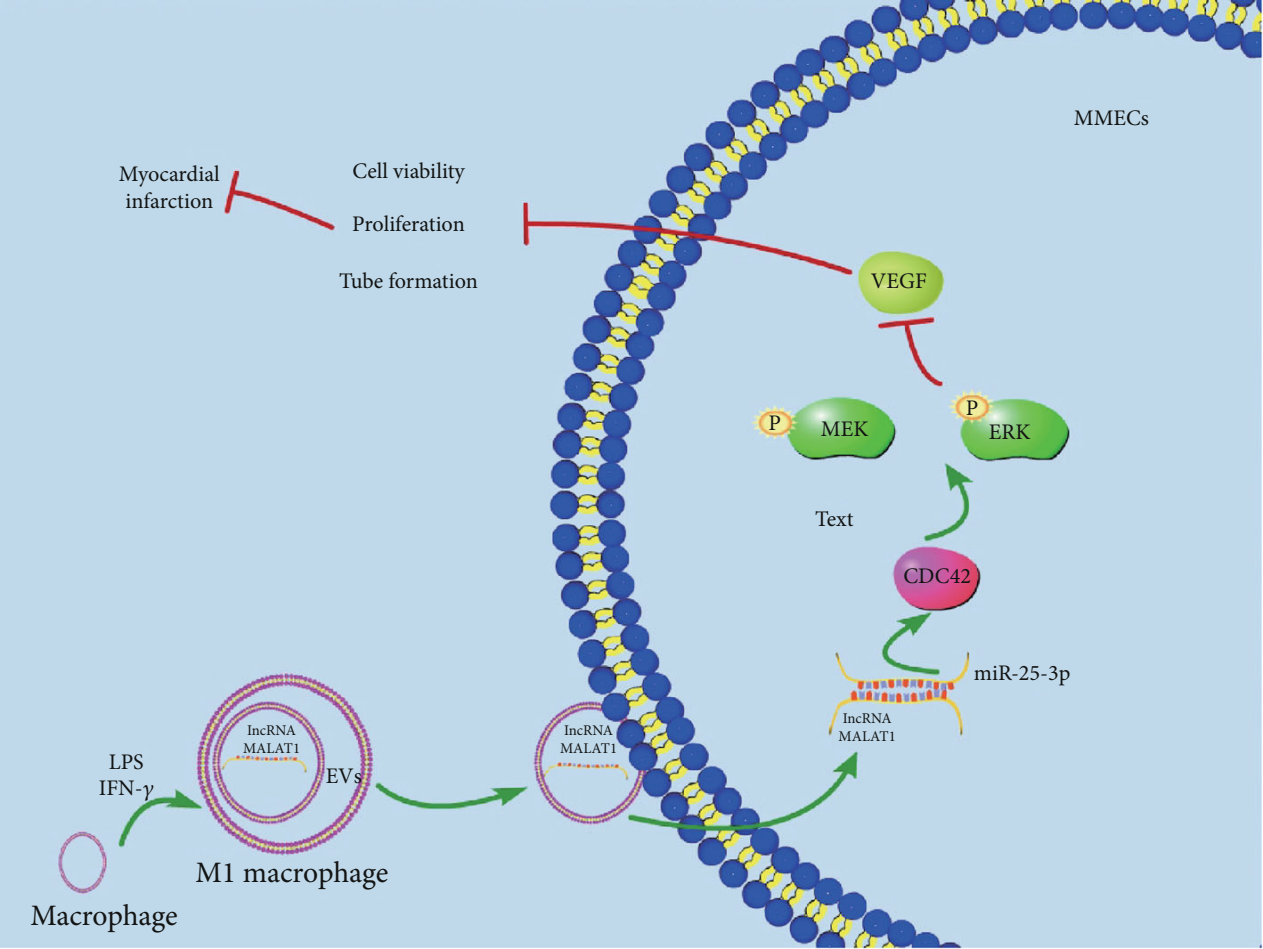
The mechanism of M1-BMMs-EVs carrying lncRNA MALAT1 to aggravating MI. LPS and IFN-*γ* induced the transformation of macrophages into M1 macrophages. M1-EVs carried lncRNA MALAT1 into myocardial microvascular endothelial cells, upregulated the expression of MALAT1 in MMECs, promoted the expression of CDC42 through competitive binding to miR-25-3p, activated the MEK/ERK pathway, and inhibited the expression of VEGF, thus declining cell activity, cell proliferation, and tube forming ability and aggravating MI.

MI results in a majority of deaths globally [47]. The inflammatory macrophages participate in the pathogenesis of MI [12]. MI seriously affects human life quality and span due to its high incidence and mortality, which brings huge challenges and stress to the global medical system [4–7]. Macrophages are the core cell population regulating the inflammatory response and have vital effects on the ventricular remodeling process after MI [14–16]. EVs are involved in the pathophysiological process of cardiovascular diseases including MI [22]. M1-BMMs-EVs aggravate the vascular injury by promoting vascular smooth muscle cell migration and proliferation in the atherosclerotic process [24, 25]. In this study, we illustrated that M1-BMMs-EV-carried MALAT1 competitively bound to miR-25-3p to promote CDC42 expression and then activated the MEK/ERK pathway, thereby inhibiting angiogenesis and myocardial regeneration following MI (Figure 10).

The crucial roles of macrophages in cardiovascular diseases, including MI, have been reported [14, 48]. M1-BMMs are associated with adverse left ventricular remodeling following MI [49]. Macrophages can secrete EVs, and macrophage-derived EVs play great roles in cardiovascular structures [50]. Therefore, we first isolated and identified M1-BMMs-EVs. Next, we turned to explore the effects of M1-BMMs-EVs on MI. Cardiac fibrosis is tightly implicated in the pathogenesis of MI [51]. According to our results, M1-BMMs-EV treatment aggravated the cardiac dysfunction and fibrosis and increased the infarct area of MI mice. M1-type macrophage-derived EVs aggravate neointimal hyperplasia following carotid artery injuries [25]. Consistently, a previous study has reported that M1-type macrophage-derived EVs facilitate heart dysfunction [52]. From all above, we proved that M1-BMMs-EVs aggravated MI in mice.

Emerging studies have provided evidence of the regulation of angiogenesis and myocardial regeneration in cardiac repair after MI [53, 54]. CD31 and VEGF have been validated as potent hallmarks for angiogenesis [55]. Our results elicited that EV treatment notably increased apoptotic cells in the myocardial infarct area and reduced CD31 and VEGF expressions. Similar results were also found in the *in vitro* experiments, as manifested by the inhibited viability and proliferation of OGD-treated MMECs and reduced VEGF expression and angiogenesis. Likewise, M1-macrophage-derived EVs inhibit the angiogenesis of endothelial cells and restrain cardiac repair, thereby aggravating MI injury [24]. Altogether, we confirmed that M1-BMMs-EV treatment inhibited angiogenesis and myocardial regeneration after MI.

Subsequently, we sought to investigate the underlying mechanism of M1-BMMs-EVs in MI. It is well accepted that EVs carry functional cargoes including lncRNAs to recipient cells to mediate intercellular communication, thus participating in the pathologies of multiple diseases [26]. In this study, we identified highly expressed MALAT1 in M1-BMMs-EVs and EV-treated MMECs. The internalization of EVs by MMECs was observed. In support of these, a previous study has proposed that EVs can transport MALAT1 to recipient cells to regulate the body energy intake [45]. MALAT1 plays a great role in cardiac remodeling and cardiovascular diseases [43, 44]. Nevertheless, little is known about the role of EV-carried MALAT1 in MI, which demonstrates the novelty of the current study. Then MALAT1 expression was downregulated in M1-BMMs to evaluate the role of M1-BMMs-EVs in angiogenesis and myocardial regeneration after MI. Our results revealed that MALAT1 knockdown promoted cell proliferation and increased angiogenesis of OGD-induced MMECs after EV treatment. Likewise, silencing MALAT1 can promote angiogenesis and repress the apoptosis of endothelial cells [56]. Briefly, we demonstrated that MALAT1 knockdown in M1-BMMs attenuated the inhibition of M1-BMMs-EVs on angiogenesis and myocardial regeneration after MI.

The downstream mechanism of MALAT1 was further explored. Through a series of database analyses and prediction, we identified that miR-25-3p was remarkably downregulated and CDC42 was upregulated in MI. Likewise, the miR-25-3p expression shows a close association with MI [57]. Abnormally upregulated CDC42 is related to myocardial injury [58]. The target relationships between MALAT1 and miR-25-3p and between miR-25-3p and CDC42 were predicted and verified. Taken together, M1-BMMs-EVs could carry MALAT1, which competitively bound to miR-25-3p to promote CDC42 expression in MI.

To verify the role of miR-25-3p in MI, we overexpressed miR-25-3p in OGD-induced MMECs. As shown by our results, miR-25-3p overexpression enhanced cell viability, proliferation, and angiogenesis of OGD-treated cells. In support of these, exosomal miR-25-3p overexpression in cardiomyocytes alleviates MI [30]. Briefly, miR-25-3p overexpression reversed the inhibitory effect of EVs on angiogenesis and myocardial regeneration following MI.

The downstream pathway of CDC42 was further explored. A previous study has proposed that CDC42 may activate the downstream MEK/ERK pathway [59]. According to our results, the MEK/ERK pathway was activated in MI, which was inhibited after MALAT1 knockdown or miR-25-3p overexpression. Consistently, the MEK/ERK pathway is activated in MI [46, 60]. Moreover, EVs can trigger the activation of the MEK/ERK pathway in gastric cancer cells [61]. Briefly, we verified that M1-BMMs-EV-carried MALAT1 activated the MEK/ERK pathway in MI.

All in all, this study supported that M1-BMMs-EVs inhibited angiogenesis and myocardial regeneration following MI via the MALAT1/miR-25-3p/CDC42 axis and the MEK/ERK pathway activation. However, studies are reporting that MALAT1 is beneficial to angiogenesis and myocardial regeneration. Hence, the role of MALAT1 in MI needs further discussion. In addition, we simply verified the activation of the MEK/ERK pathway. In the future, we will further study the specific mechanism of the MEK/ERK pathway and verify whether MALAT1 can be used as the entry point of MI management.

## Figures and Tables

**Table 1:** Primer sequences for RT-qPCR.

Gene	Primer
MALAT1	F: 5′-ATGAGCCAGAGATTGTTCCTACTG-3′
	R: 5′-TTAGCTATCCCATCATGAAAGCC-3′

miR-96-5p	F: 5′-TTTGGCACTAGCACATTTTTGCT-3′
	R: 5′-GCAAAAATGTGCTAGTGCCAA-3′

miR-92a-3p	F: 5′-TATTGCACTTGTCCCGGCCTG-3′
	R: 5′-CAGGCCGGGACAAGTGCAA-3′

miR-124-3p	F: 5′-TAAGGCACGCGGTGAATGCC-3′
	R: 5′-GCATTCACCGCGTGCCTT-3′

miR-92b-3p	F: 5′-TATTGCACTCGTCCCGGCCTCC-3′
	R: 5′-GGAGGCCGGGACGAGTGCAA-3′

miR-582-5p	F: 5′-ATACAGTTGTTCAACCAGTTAC-3′
	R: 5′-GTAACTGGTTGAACAACT-3′

miR-25-3p	F: 5′-CATTGCACTTGTCTCGGTCTGA-3′
	R: 5′-TCAGACCGAGACAAGTGC-3′

U6	F: 5′-GTGCTCGCTTCGGCAGCACATATA-3′
	R: 5′-AATATGGAACGCTTCACGAATT-3′

GAPDH	F: 5′-ATGCTGCCCTTACCCCGGGGTCC-3′
	R: 5′-TTACTCCTTGGAGGCCATGTAGG-3′

## Data Availability

All the data generated or analyzed during this study are included in this published article.
